# Every story has two sides: evaluating information processing and ecological dynamics perspectives of focus of attention in skill acquisition

**DOI:** 10.3389/fspor.2023.1176635

**Published:** 2023-05-24

**Authors:** Victoria Gottwald, Marianne Davies, Robin Owen

**Affiliations:** ^1^ Department of Sport and Exercise Sciences, Bangor University, Bangor, United Kingdom; ^2^ Sport and Physical Activity Research Centre, Sheffield Hallam University, Sheffield, United Kingdom; ^3^ School of Health and Sport Sciences, Liverpool Hope University, Liverpool, United Kingdom

**Keywords:** sport, attentional focus, movement, cognition, dynamical systems, motor learning

## Abstract

Directing our focus of attention appropriately during task execution can benefit outcome performance, cognitive efficiency, and physiological efficiency. For instance, individuals may benefit from adopting an external focus of attention (i.e., by focusing attention on the effects of one's movements on the environment) over an internal focus of attention (e.g., focusing on one's body movements). However, accounts concerning the theoretical functioning of such effects have primarily relied on hierarchical information processing perspectives; far less consideration has been given to potentially alternative explanations based on ecological dynamics, instances where an internal focus may be desirable over an external focus, and the associated applied implications. Within the present review, we: (a) outline the most recent developments in attentional focus research; (b) evaluate similarities and differences between information processing and ecological dynamics explanations of the focus of attention effect; (c) provide practical recommendations; and (d) discuss future research avenues. In doing so, a case is made for an “Ecological Dynamics Account of Attentional Focus” to act as an alternative to information processing-based hypotheses.

## Introduction: the focus of attention phenomenon

Verbal instruction is one of the most common methods of conveying information to individuals when learning and performing motor skills. However, it is now well established that the language we use when providing instruction can influence the skill acquisition process, particularly in relation to whether it directs an individual's attention internally towards the body or externally towards the effect of one's movements on the environment (1). This phenomenon is consistent with early rhetoric from James (2) when discussing the influence of attention on movement outcomes: “Keep your eye at the place aimed at, and your hand will fetch the target; think of your hand, and you will likely miss your aim” (p. 520). There is now a wealth of literature supporting an external focus of attention for several performance outcomes; including accuracy, speed, cardiovascular endurance, maximum force production, movement kinematics and motor economy [for reviews see (1, 3)]. Benefits of an external focus also extend beyond sport and have been applied to enhance movement solutions within varying domains, including the military (4) and healthcare fields. Examples include when working with Parkinson's (5), stroke (6), or multiple sclerosis patients (7), as well as those with intellectual disabilities (8), in older populations (9), requiring falls prevention (10), and in rehabilitation environments such as individuals recovering from ankle sprains (11) or ACL reconstructive surgery (12).

**Figure 1:**
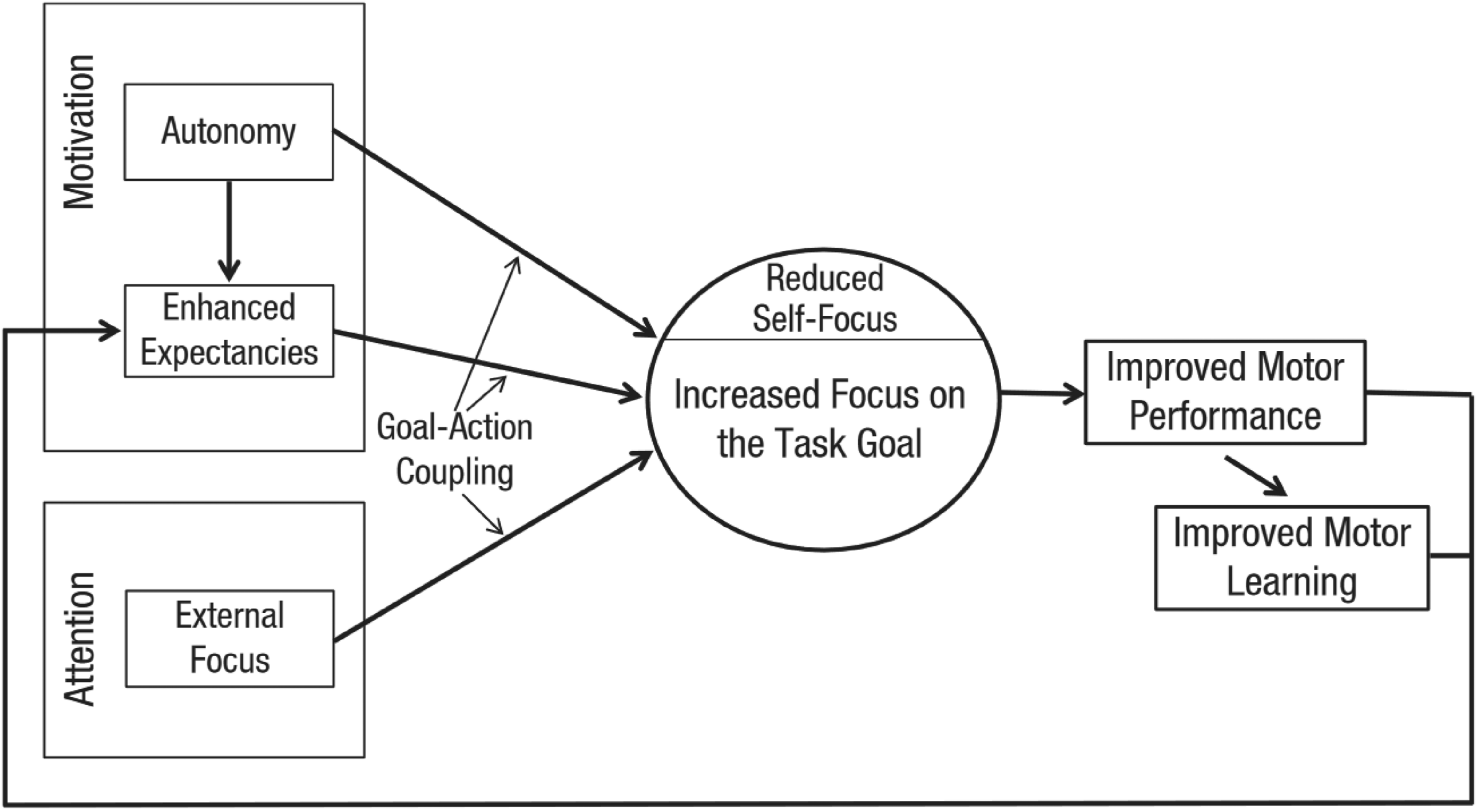
Schematic depicting Wulf and Lewthwaite's (24, 25) OPTIMAL theory of motor learning.

**Figure 2:**
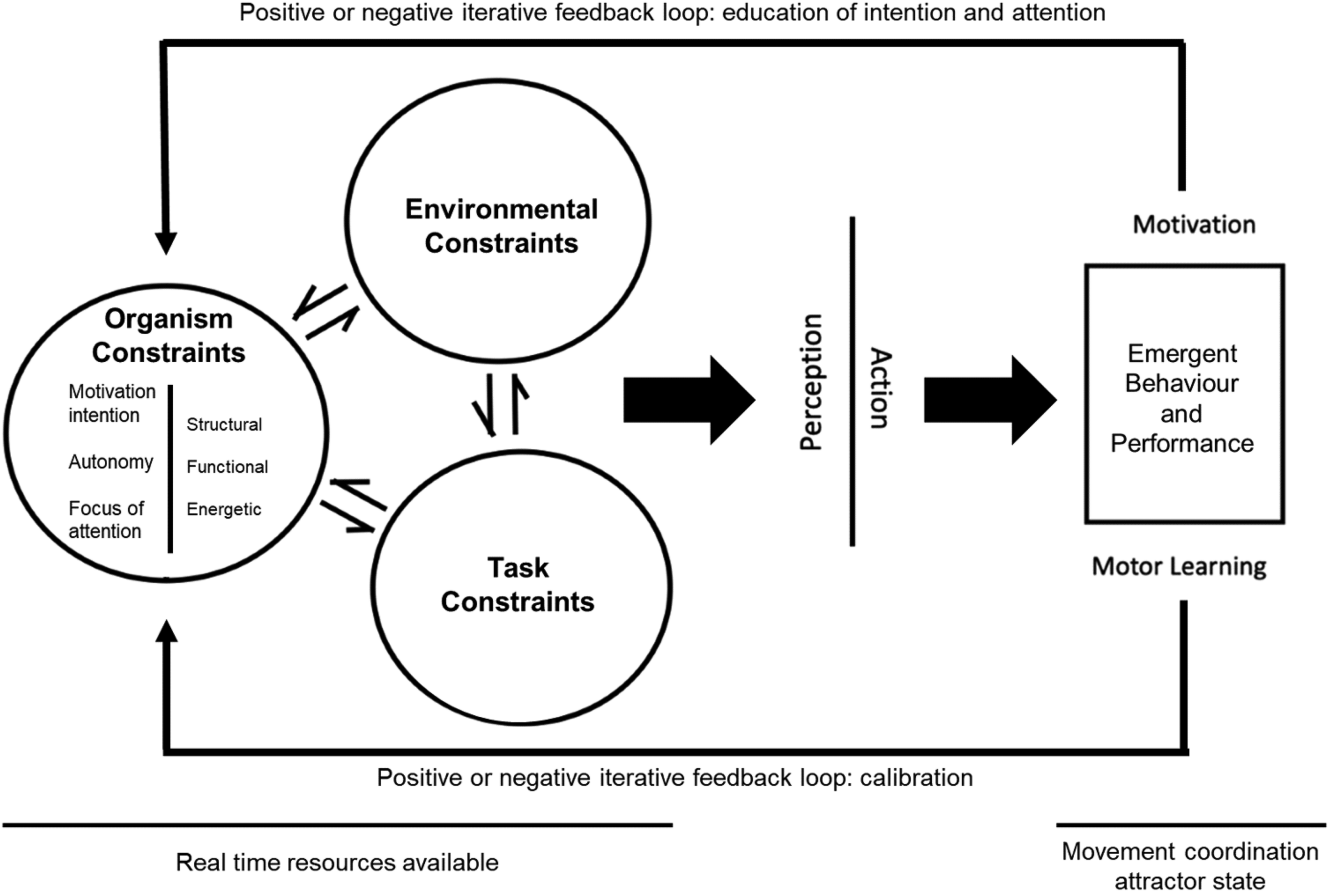
An ecological account of focus of attention, adapted from Davies & Davies (105), Newell's (83) constraints model, and Wulf and Lewthwaite's OPTIMAL model (24, 25).

However, accounts concerning the theoretical mechanisms underpinning such effects have primarily relied on hierarchical information processing perspectives, whilst far less consideration has been given to alternative explanations based on ecological dynamics and the applied implications thereof. There remains debate in the skill acquisition field with regards to the extent to which end-uses of theory (i.e., practitioners such as sports coaches) should comprehend the theoretical underpinnings of skill learning so that they may best align practice decisions with their chosen perspective. Philosophically, if we adopt a “shed-building metaphor”: one belief is that if you build a shed in your garden, whether you believe the earth is flat or spherical, has little influence on the way in which the shed is built. However, there should be little doubt that for the best practice conditions to occur, coaches must be able to justify their decision making and articulate the rationale underpinning applied practice decisions. This has implications on coach education, where adequate skill acquisition and pedagogical training is arguably sporadic on a global scale (13).

The present review aims to address these issues by providing greater clarity in relation to the fundamental theoretical principles underpinning differing perspectives that account for the focus of attention effect within skill learning/performance, and aim to address implications for applied practice. More specifically, the review will: (a) outline the most pertinent developments in the attentional focus literature; (b) evaluate similarities and differences between information processing and ecological dynamics explanations of the focus of attention effect; (c) provide practical recommendations and suggestions for coach education; and (d) discuss future research avenues. In doing so, a case is made for an Ecological Dynamics Account of Attentional Focus to act as an alternative to information processing-based hypotheses.

## Recent research directions

### Focus distance

When selecting appropriate external foci, some contexts require practitioners to decide between multiple alternatives. For example, a hockey coach choosing to direct attention towards the club vs. the ball, or a medical doctor choosing to attend to their scalpel vs. the target epidermis. This conundrum has led researchers to investigate “the distance effect”, whereby benefits of distal external foci (i.e., environmental/task information far from the body) over proximal external foci (i.e., environmental/task information close to the body) were first identified by McNevin et al. (14), on the basis that attending to external movement effects further from the body are more easily differentiated from the body and thus more likely to facilitate automaticity. At first glance, this finding seems robust across experts and novices (14–16), but more recently, Singh and Wulf (17) have reported some interesting nuances. Whilst the authors provide further evidence to support a more distal focus for the expert performer, for tasks that require coordination of greater degrees of freedom (e.g., a snatch in weightlifting), then it is argued that a proximal external focus that is better aligned with technique, may be more appropriate for novices. Differential findings as a function of expertise were also supported with measures of focus preference (i.e., experts preferred a distal focus and novices more proximal). Singh et al. (18) have accounted for these findings with the notion of functional variability when distality of focus is appropriately matched to expertise level. The authors showed evidence for enhanced coordination of the shoulder, elbow and wrist in a volleyball serve, for skilled performers adopting a more distal as opposed to proximal focus.

Interestingly, the distance effect has also been considered in the context of an internal focus. Pelleck and Passmore (19) investigated a range of performance metrics when adopting an internal attentional focus more proximal or distal to technical features of the task. The authors hypothesised that the detrimental effects of an internal focus would be exacerbated when more proximal to critical elements of the technique, by crating greater interference with automatic self-organisation thereof. Indeed, in a golf putt, measures of movement accuracy, muscle activity, and kinematics were all adversely affected when directing attention proximally towards technique-relevant upper-body as opposed to distally towards technique-irrelevant lower-body limb mechanics.

### Focus relevance

Findings from Pelleck and Passmore (19) suggest that any disturbances to the motor system when focusing internally, may be concentrated towards more skill-relevant bodily factors in tasks otherwise reliant on environmental afferent information. It is conceivable that focusing internally on limb mechanics which are responsible for action (e.g., upper-body in golf) may be of least task relevance in far-aiming tasks, since the motor system is capable of self-organising limb mechanics without a need for conscious monitoring [see also (20)]. In this manner, it is possible that focus relevance may moderate the relationship between expertise and focus distance (17, 21). Indeed, Amini and Vaezmousavi (4) reveal enhanced shooting performance in elite military personnel when adopting a more task relevant external focus (regardless of distality) [see also (22)]. An external-relevant focus which comprised mentally focusing on the target facilitated superior shooting performance compared to an external-irrelevant focus which comprised focusing mentally on a randomly presented auditory stimulus to judge its bass and treble. Therefore, relevance of the external focus to the task may be a key consideration when formulating instruction.

### Focus salience

With focus relevance intricacies in mind, recent research has emphasized the complexities of selecting appropriate external foci for learning and performance. Mechanistic explanations underpinning the attentional focus phenomenon have tended to emphasize the role of an external focus to augment congruence between planning and action, and ultimately enhance automaticity of motor programming (see Wulf et al.'s (23)], constrained action hypothesis, and Wulf & Lewthwaite's (24, 25) notion of goal-action coupling, respectively). However, it stands to reason that these mechanisms rely on there being a more tangible (i.e., relevant) movement effect such as motion of a club, racket, or ball. In the absence of this, Lawrence et al. (26) argue that the benefits of an external focus may be diminished. Whilst there remain some inconsistencies in the literature when investigating this type of form task [see also (27)], there is little doubt that from a practitioner perspective, some tasks may exhibit challenges when identifying more salient external movement effects e.g., within floor gymnastics or dance. More recently, Becker et al. (28) present a novel solution to such instances, via a holistic attentional focus that is targeted towards generalised feelings of the movement to inhibit conscious control of effectors. When tested in a standing long jump, findings revealed that both an external and holistic focus enhanced performance, with no statistical difference between the two. Becker and colleagues advocate a holistic focus when an external focus is neither practical nor desired. A similar solution which adopts what the authors term a “mind over body” approach, entails replacing body parts (in this instance, the supine forearms in a volleyball pass), with the depicted image of an external object (a “platform”) (29). This shows promising findings and is consistent with more traditional implicit learning techniques such as analogy learning (30).

### Wider psychological mechanisms

Irrespective of nuances surrounding the distance effect and skill relevance, the literature to date presents a robust representation of the attentional focus phenomenon, and benefits of an external focus. However, this literature has typically considered the relationship between small numbers of variables in isolation, for example the influence of an external focus of attention on electromyography (EMG) or movement amplitude, somewhat removed from interactive psychological functioning (see 1 for a review). These linear methodologies have justifiably been adopted in the name of conserving methodological integrity and rigour. However, a more recent research direction has begun to embrace more holistic and non-linear methodologies, arguably more consistent with skill acquisition in practice. In this manner, Wulf and Lewthwaite's (24, 25) OPTIMAL theory (optimising performance through intrinsic motivation and attention for learning) (see Figure 1), proposes that learning is a consequence of interactions between both attentional and motivational factors. The authors speculate that the ideal sensorimotor and motivational conditions can lead to enhanced goal-action coupling via use of more efficient functional connections across brain networks. Specifically, learning environments which promote autonomy (e.g., choice in the training activity undertaken) and enhanced expectancies (e.g., belief that the training activity will benefit performance) should increase dopamingergic responses and engagement with the task, which when combined with an optimal external attentional focus direction, enables individuals to achieve greater neural coupling between the task goal and action being organised. Therefore, wider psychological mechanisms may be a valuable consideration in focus of attention literature and applied practice going forward.

### Ecological validity

The shift in research direction to investigate the attentional focus phenomenon more holistically, has also cemented a need to test in in more ecologically valid environments. Despite an extensive literature-base supporting the robustness of an external focus of attention to enhance a breadth of movement outcomes (see 1 Wulf, 2013), the field has arguably failed to bridge the gulf between theory and practice. There remain significant discrepancies between what is advocated by empirical research and the language being observed from coaches and practitioners in the field (31). Research methodologies embracing the value of investigating attentional focus in more “naturalised” environments, are likely to give us a better understanding of the “what, when, why, and how” of different focus instructions and strategies, and subsequently identify why these discrepancies exist so that we might ensure efficacy of coach education. Whilst several studies have now adopted observational approaches to identify the nature of attentional focus instructions and strategies employed in sport and rehabilitation environments [e.g., (32–35)], richer qualitative approaches [e.g. (36)], have advanced this further to: (a) explore the functionality of different focus instructions across both practice and competition environments; (b) investigate differences in attentional focus across different aspects of the game i.e., the short vs. long game; and (c) identify the mechanisms influencing adoption of attentional foci, e.g., self-generated vs. coach-led instruction. Findings highlight the complexities underpinning the attentional focus phenomenon and likely account for discrepancies between research and practice. For example, whilst coaches had a role in influencing the attentional focus adopted in elite-level golf, there was a lack of consistency between the attentional focus advocated by coaches and what was adopted by players in practice and competition. Furthermore, the attentional focus adopted by players varied between the short- and long-game, with players more likely to focus on the body during the short-game, and focus during competition environments typically being self-generated by players as opposed to coming from the coach. Isolated coach education interventions are therefore unlikely to be sufficient in enhancing the extent to which an external focus is employed during practice environments.

When investigated in more ecologically valid settings with athletes, Anderson et al.'s (37) findings are consistent with the notion that the attentional focus effect is likely more complex than is currently portrayed by the literature. The authors adopted machine learning techniques to identify patterns of attributes that differentiated between two groups of athletes: high and low performing Olympic weightlifters. Associated odds ratios revealed that athletes were 9.5 times more likely to achieve high-performing status if they had completed over 281 h of practice using an internal focus of attention by the first phase of testing. It is important to note however, that whilst this was the case, athletes were also 9.3 times more likely to reach the same status if they had completed over 346 h using an external focus of attention by the same stage. Together, these findings suggest that different types of focus instructions might possess different functions during an athlete's development.

### Facilitative somaesthetic awareness

Similarly to the complexities associated with ecological settings, the purpose of focus of attention prescription also appears to play an important role in determining optimal attentional foci. Toner and Moran (38) propose a functional “somaesthetic awareness” for correcting bad habits. They advocate switching between what they term more reflective (internal) and unreflective (external) modes of bodily awareness, the same way in which an athlete might switch between the autonomous and associative phases of learning (39) when making adjustments to problematic movements that would normally be executed outside of conscious control. This is also consistent with Carson and Collins’ (40–42) non-linear Five-A model of technical refinement [analysis, awareness, adjustment, (re)automation, and assurance], wherein the process of skill refinement is differentiated from that of skill learning. The second stage of the process centres around “awareness” with the authors arguing that the skill must be “de-automised” prior to technical corrections being made. More recently, Gottwald et al. (43) suggest that an internal focus (or somaesthetic awareness) may also have value when congruent with afferent information more useful for task success e.g., proprioceptive tasks such as artistic gymnastics. This was tested over a series of three studies using upper and lower limb extension tasks, where pertinence of proprioceptive information was enhanced by removing vision or adding weighted objects to limbs involved in movement production. Enhanced movement economy via reduced EMG activity was consistent with outcome measures of performance accuracy when adopting a congruent internal focus. These findings warrant further investigation in more ecologically valid tasks but may account for the incongruous findings in Olympic weightlifting (37), where proprioception is arguably integral to successful movement execution of the snatch and clean and jerk. In a similar task, Kal et al. (44) also revealed trends supporting enhanced automaticity for stroke patients when adopting an internal focus. The authors accounted for these findings with the notion that this population may have preferred using an internal focus in daily life, perhaps strengthened by familiarity as inferred from Collins et al. (45).

This notion of a facilitative somaesthetic awareness is also supported by Moore et al. (46), who investigate the value of using different attentional focus prompts in rearfoot-striking runners, to correct problems in their gait and achieve a flatter foot at ground contact. An internal focus was shown to be more effective for retraining kinematics with no detriment to physiological responses. Similarly, Schücker et al. (47) showed that focusing on the feeling of the body in endurance running did not disrupt movement economy if the focus was not directed towards a highly automated process such as breathing. This has implications for use of an internal focus for pacing. Similarly, Neumann et al. (48) have revealed benefits of an internal focus in rowing where performance outputs were not constrained. Participants focusing on a series of internal vs. external cues, showed performance benefits via distance rowed, power output per stroke and physical exertion. These complexities are consistent with the notion that internal and external foci might be more appropriate for different functional roles. Recent evidence (49, 50) suggests that switching attention between movement preparation and execution might benefit performance. This is also supported by Gottwald et al. (43) who identified benefits of an internal focus for motor planning, but not control, in proprioceptive tasks.

## Focus of attention from an information processing perspective

Accounts concerning the theoretical functioning of the attentional focus effect have arguably been skewed towards hierarchical information processing perspectives, wherein movement plans are purportedly stored in memory and transmitted to the limbs for execution (51). Cognitive, or “information processing” accounts of motor learning, adopt the standpoint that the brain is a metaphoric “computer”, processing sensory inputs prior to providing an output in the manner of an appropriate motor response (52). This theoretical perspective relies heavily on schema theory (53, 54), which proposes that the general characteristics of actions (i.e., relative timing and force) are represented cognitively in memory and can be drawn upon for motor execution when required. Different states of memory, or “schemas” have responsibility for different processes, with the recall schema responsible for movement production and the recognition schema responsible for movement evaluation, allowing for error detection and correction. Whilst some features of Schmidt's original (53) motor schema theory have been contradicted empirically in the literature [see (54)], the primary tenet of information processing accounts of motor learning, which still stand today, supports the notion that actions are “pre-programmed”, a direct contradiction to mechanisms underpinning ecological dynamics frameworks.

Wulf et al.'s seminal (55) series of studies, which were arguably the impetus for the attentional focus research, first accounted for the benefits of an external focus in a ski-simulator and balance task, with ideomotor-based principles of motor learning [see (56)]. Whilst traditional information processing models present a certain dissociation between perception and action (i.e., input and output), ideomotor principles propose that actions are indeed represented in the brain but in relation to their anticipatory sensory consequences. Prinz's (57) common-coding theory proposes a shared coding system for perception and action. In line with this, Prinz's action-effect principle suggests that “actions are planned and controlled in terms of their effects” (p. 152). Wulf et al. suggested that providing (external) instructions that direct attention towards the effects of one's movements on the environment, only serves to augment the intrinsic association between afferent and efferent information and enhance skill learning. If actions are “coded” in line with their movement effects, then it stands to reason that adopting an internal focus of attention will likely inhibit automaticity of response programming.

Wulf and colleagues (23) tested this hypothesis in a balance task, where participants had to respond to an auditory tone by pressing a button as fast as possible whilst balancing under either internal or external focus conditions. As hypothesised, an external focus of attention facilitated automaticity of the motor system, evidenced by faster probe reaction times combined with enhanced balance performance. These findings led to the conception of what is now well established in the literature as the “constrained action hypothesis”. Specifically, Wulf et al. proposed that an internal focus directs conscious attention to otherwise automatic movement processes, that operate more efficiently and effectively if left unattended via an external focus. These mechanisms have since been supported rigorously with various neurophysiological and kinematic measures, including electromyography (EMG), electroencephalography (EEG), and movement variability (58). More specifically, reductions in muscular activity via EMG support the notion of increased movement economy when using an external focus (59) and this effect has now been replicated in dynamic tasks such as jumping (60) or shooting in basketball (61), as well as more static tasks where EMG data is arguably more stable [e.g., within isometric force production; (62)]. Parr et al. (20) extended this by testing EMG together with EEG, during an isometric upper limb force precision task to better understand neuromuscular control as a function of attentional focus. Findings were consistent with previous literature, with the forearm flexor showing greater EMG activity when using an internal focus but also increased EEG alpha activity across the parieto-occipital cortex, a possible indication of increased conscious processing. Support for enhanced cortical processing has also been corroborated with measures of movement planning. Data suggests that an external focus may facilitate offline planning efficiency via reduced pre-movement times in an isometric force production task (63). This is further evidence for increased automaticity and reduced conscious processing. Furthermore, and not surprisingly, these neurophysiological benefits seem to result in more optimal movement kinematics. For example, Lohse et al. (64) showed evidence for increased variability (standard deviation) at the shoulder joint upon extension, when adopting an external focus of attention in a darts throw. This “functional variability” is consistent with Bernstein's (65) degrees of freedom problem, which proposes that movements are only constrained to the point where functionality is optimised.

Wulf and Lewthwaite (24, 25) have since tried to consider these attentional mechanisms in conjunction with psychological factors underpinning motor learning, on the basis that the role of motor, social, cognitive, and affective mechanisms should be considered as complex interactions in line with human function, and not in isolation. Specifically, OPTIMAL theory proposes that adopting an external focus of attention in conjunction with autonomy and enhanced expectancies for success, stimulates advantageous dopamine responses, augmenting “goal-action coupling”. This is based on the notion that learners working in these sensorimotor and motivational environments will become more focused on their task goals and direct focus away from the self. Wulf and Lewthwaite speculate that this can result in a continuous cycle of enhanced motor learning, whereby an external focus of attention combined with enhanced expectancies for success results in not only successful movement outcomes, but also increased levels of self-efficacy and positive affect, which in turn influence perceived competence and so the cycle continues. However, early empirical tests of OPTIMAL theory, provide equivocal support for this framework [e.g., (66–68)]. Simpson and colleagues (67) revealed that although an external focus, led to enhanced motivational states (i.e., self-efficacy, perceived competence, task effort, and positive affect), integrating attentional focus with conditions that enhanced expectancies for success did not provide additional motor-performance benefits over and above an external focus alone, in a standing long-jump task. Further research testing the complex interactions between attentional and psychological mechanisms is warranted.

## The ecological dynamics account of attentional focus

Ecological dynamics is underpinned by the interlinking of dynamical systems theory and ecological dynamics, focusing on the individual-environment relationship mediated through perception-action coupling (69–72). Rather than a linear top-down control of movement, it is the interaction of intention and the information perceived in the environment that controls movement. Consequently, perception and movement are inextricably entwined and cannot be separated. The ecological dynamics approach to motor learning posits that, instead of movement plans being stored in memory and called upon when needed, movement is continuously (re)organised based on the dynamical interaction between organism, task, and environmental constraints.

Individuals' direct perception of the situational opportunities for action (i.e., affordances) in relation to their organismic, environmental, and task constraints, enables them to dynamically self-organise movement coordination into stable states (i.e., attractors), which achieve the desired goal. Consequently, an ecological dynamics framework features greater explanatory power than information processing accounts, with regards to individuals' functional adaptability within a world high in degrees of freedom (72, 73). For example, even in the most exceptional of circumstances when playing soccer (e.g., opposition players obstructing the field of vision, heavy rain, uneven pitch terrain, and temporarily reduced range of movement because of injury), players can still exhibit capacity for successful passes. For both novelty and storage reasons, information processing-based mechanisms are less able to account for such instances than ecological dynamics. Given the explanatory power of ecological dynamics and its growing prominence within motor learning (74), it is timely to develop an “Ecological Dynamics Account of Attentional Focus”.

Firstly, the action-effect principle of common coding (57) and the goal-action coupling of OPTIMAL theory (24, 25), account for attentional focus effects by suggesting that movements should be planned in relation to their intended effects/goal for optimal parameter selection; this seminal work predominantly assumes that an external focus most closely aligns with intended effects/goals in all tasks, since actions take place in the external environment (1, 24, 25, 55). However, the presently proposed Ecological Dynamics Account of Attentional Focus (see Figure 2) offers a more nuanced explanation for common coding and goal-action coupling effects, based on direct perception. This concept proposes that individuals do not perceive the world in terms of absolute physical parameters (e.g., speed and angle of a player) but instead in terms of affordances [e.g., whether the player can be tackled; (75, 76)]. Essentially, the external environment is directly perceived in proportion to the organism's intention and internal bodily motor capacities. Within this framework of perception, cognition plays the role of a supervisor (77, 78) and distributes the organism's limited resources for the perception of information across the body and environment (i.e., specifying information for the constraining of action); thus, adopting a specific focus of attention may be a product of cognition's attempt to distribute limited attentional resources to specifying information deemed most relevant. Similar to previous common coding and goal-action coupling accounts, it can be assumed that within far-aiming tasks [e.g., golf (16)], an external focus of attention on environmental specifying information such as the target, may identify more desirable opportunities for action (affordances) and allows individuals to organise into more accurate and efficient attractor states which hit the target. Relatedly, focus of attention distance (distal vs. proximal) and task relevance (relevant vs. irrelevant) effects may be a product of limited attentional resources being allocated to more vs. less useful specifying information when determining affordances for action. For example, in a far aiming task such as a golf putt, a proximal external (e.g., focus on the club) may provide less valuable specifying information than an external distal (e.g., focus on the ball's trajectory into the target hole), since the latter aligns closer with intention. However, converse to previous common coding and goal-action coupling accounts, within form tasks [e.g., gymnastics: (26)] and proprioceptively guided tasks [e.g., Olympic weightlifting: (37)], an internal focus of attention on bodily specifying information (e.g., arm straightness), may likewise result in the identification of more accurate and efficient attractor states which achieve superior form/technique required by the task. Essentially, tasks guided by environmental specifying information may benefit from an external focus on relevant aspects in the environment, while tasks guided by bodily specifying information may benefit from an internal focus on relevant aspects concerning the body. Therefore, an Ecological Dynamics Account of Attentional Focus features explanatory power across a wider range of foci (i.e., including instances where internal foci yield superior performance) compared to previous common coding and goal-action coupling accounts.|

Secondly, the constrained action hypothesis (23) suggests that an external focus of attention facilitates movement accuracy, physiological efficiency, and cognitive efficiency by directing individuals' attention towards external environmental aspects, which proposedly do not consciously interfere/constrain the motor system's ability to self-organise. However, as identified by Davies (79), the constrained action hypothesis account of external focus effects is already closely aligned with ecological dynamics. The Ecological Dynamics Account of Attentional Focus would predict that directing attention to situationally relevant specifying information would facilitate emergent self-organisation in relation to intention. An internal focus of attention in tasks guided by external environmental specifying information may result in the use of less relevant bodily specifying information for natural self-organisation processes. This may result in reduced accuracy and physiological efficiency via misinformed attractor states, as well as reduced cognitive capacity via inefficient use of attention by needing to evaluate task-essential environmental specifying information while also consciously monitoring less relevant bodily specifying information via an internal focus. Instances of external foci in tasks guided by bodily specifying information may follow a similar pattern. Unlike the constrained action hypothesis, such processes would explain the performance and efficiency benefits when adopting: (a) an external focus of attention in primarily external-information-reliant far aiming tasks [e.g., (61)]; (b) an internal focus of attention in primarily internal information-reliant form or proprioception tasks [e.g., (43)]; and (c) more vs. less task-relevant versions of either focus of attention [e.g., (4, 22, 19)]. For example, in situations where proprioceptive information is paramount for task success, an external focus of attention may direct conscious attention to task-irrelevant environmental constraints; thus, reducing accuracy and efficiency of actions whilst also increasing attentional load.

Lastly, OPTIMAL theory of motor learning posits that adopting an external focus of attention in conjunction with an appropriate motivational climate (i.e., enhanced expectancies and autonomy) augments the “goal-action coupling” (24, 25). Within the Ecological Dynamics Account of Attentional Focus and in line with ecological psychology, the education of intention (e.g., motivation via autonomy and enhanced expectancies), education of attention (e.g., increased sensitivity to specifying information), and the calibration of perception and action sub-systems is assumed to facilitate perception of energy (e.g., wind, object momentum, or ground composition) as lawfully structured specifying-information, which is direct and functionally meaningful to the organism without interpretation (69, 70). Based on this, it is possible to reinterpret the central “goal-action coupling” of OPTIMAL as the use of appropriate specifying information to facilitate perception of detailed and relevant interactions between environmental, organismic, and task constraints (80). Of note is that the organism may indeed have their own motivational (intention) and focus of attention (attentional) constraints, which affect optimal functioning and computation of structured energy as functional specifying information. Motivational constraints may influence the organism's ability to identify affordances (opportunities for action) that align with their goals, sustain task-relevant attention, or inhibit task-irrelevant distractions. Focus of attention constraints may influence whether individuals pick-up or become sensitised to task-relevant specifying information. These constraints interact with environmental (e.g., opposition player location) and task (e.g., environmental external vs. proprioceptive internal) demands to inform perception and guide self-organisation into stable attractor states for action. Through interaction with tasks and the environment, feedback loops in response to action would subsequently lead to adaptations of the organisms' constraints via education and calibration of perception, ultimately influencing action going forward. These changes are more akin to tuning a radio set to be more sensitive to picking up desired frequencies, than changes in a computer programme made by a programmer. As with more modern and high-tech radios, the changes influence the organism-environment/task relationship (what can be perceived), not what is stored inside the organism. Learning is a gradual process of becoming attentive to, and interested in, what is going on around us. It is a process that requires us to learn to attend to things, rather than acquiring the knowledge that absolves us of the need to do so (81). Consequently, behaviour emerges through the coupling of movement to perceptual information due to the self-organisation of the movement degrees of freedom.

To summarise the aforementioned sections, the Ecological Dynamics Account of Attentional Focus has three core tenets. Firstly, to facilitate optimal perception for action, the direction of the attentional focus needs to be congruent with task demands and their most relevant specifying information. Tasks guided by external environmental specifying information may exhibit superior self-organisation via an external focus on relevant aspects in the environment, while tasks guided by internal bodily specifying information may exhibit superior self-organisation via an internal focus on relevant aspects concerning the body. Secondly, utilisation of foci incongruent with task demands may result in the use of less relevant specifying information for natural self-organisation processes; this may result in reduced accuracy and physiological efficiency via misinformed attractor states, as well as reduced cognitive capacity via inefficient use of attention which needs to evaluate task-essential specifying information while also consciously monitoring less relevant specifying information derived from the adopted focus. Thirdly, it is possible to reinterpret the central “goal-action coupling” of OPTIMAL theory as the identification of appropriate specifying information from the structured energy comprising the world, to facilitate perception of detailed and relevant interactions between environmental, organismic, and task constraints. This reaffirms the impetus to select an attentional focus (organism constraint) in relation to environment and task constraints. Overall, the Ecological Dynamics Account of Attentional Focus assumes that attentional focus is not one size fits all, but dependent on its suitability when combined with the interacting constraints which influence perception for action; even intra-individually as performers continuously attune to perceptual information that specifies action. This links to the ecological mantra for coaching, helping the individual to define “where to look, not what to see” (82).

Crucially, the Ecological Dynamics Account of Attentional Focus provides a novel and arguably more congruous explanation for focus of attention effects than common coding, constrained action hypothesis, and OPTIMAL theory (23–25, 55). As noted by Davids (79), the mechanistic explanations put forward by Wulf and colleagues, somewhat borrow from both information processing and ecological dynamics (i.e., constrained action hypothesis' championing of self-organisation), while also constraining themselves via assumptions of an external focus of attention's superiority in all conditions. An ecological dynamics standpoint provides a more conceptually consistent framework, as well as a more flexible account in instances where an internal focus of attention may prove desirable. This latter aspect may be in part because, from an ecological dynamics perspective, the distinction between an internal and external focus of attention is less clear-cut. An external focus of attention and its resultant benefits have long been conceptualized as focus on “movement effects” (55). However, an affordance is a relationship between an organism (internal aspects) and the environment (external aspects). From an ecological dynamics perspective, it may be beneficial to reconceptualise the beneficial effects of focusing on “movement effects” as representing focus on task-relevant aspects of performance or specifying information, rather than exclusively external information *per se*, as suggested by Herrebroden (21).

## Practical recommendations

When designing effective practice environments in relation to attentional focus, we would advise the following process is adhered to: “function before context” i.e., first consider the primary objective of the practitioner (e.g., skill learning, technical refinement, fostering movements that minimise injury risk, or developing techniques that are under robust pressure) and then consider the context in relation to the motor skill (e.g., far aiming vs. proprioceptive tasks), the relevance and proximity of possible foci (where appropriate) and the appropriateness of instructions altogether (see Table 1).

**Table 1:** Practical solutions to support applied practice.

Applied challenge/context	Applied practice solution	Practical example	Theoretical rationale	Supporting evidence
*When there is a decision to be made to select the most appropriate external focus in a skill requiring coordination of several body parts*	Proximal external focus when working with individuals in early stages of learning and distal external focus when working with individuals in late stages of learning	A novice focusing on the racquet motion during a tennis serve versus an expert focusing on the intended ball trajectory	Focusing proximally allows novices to attend to skill-relevant information and assemble optimal coordination patterns. Focusing distally promotes motor automaticity for experts. In the gaining control, during the stabilization phase, learning is focussed on attunement to specifying perceptual information which can then be exploited in the skilled optimisation phase through effective calibration of action to the perceived information	(16, 17, 84)
*When there is a need to “simulate” an external focus because this doesn’t exist naturally*	Using visual images to replace body parts Using a replacement for the missing information	Imaging a ‘platform’ in place of the forearms during a volleyball pass Using pegs to represent the position of fielders when practising in a cricket net	Prevents individuals focusing on body-centred information and constraining actions Gives a purpose to external focus when the practice is devoid of the information that would be present in a competition	(29, 30, 85)
*When a meaningful external focus cannot be easily identified*	Adopting a holistic focus of attention	Focusing on making your movement ‘feel explosive’ in a standing long jump task	Prevents individuals focusing on body-centred information and constraining actions	(28, 86–88)
*To identify clear session intentions*	Goal-orientated practice	Having outcome goals rather than movement form goals	Frames interactions with task and environment. Supports the development of picking up information and using strategies that may vary depending on individual differences and the functionality of information and movement coupling	(89)
*When the demands of competition must be matched to practice*	Representative learning design Ensuring that task-relevant information is available in practice	Designing tennis practice to contain more information that is representative of competitions such as the behaviour and intentions of opponents	Supports attunement to the information that will be present and specifying in competition	(90–92)
*When there is a need to enhance the extent to which skills are adaptable to changing environments*	Promoting self-organisation	Using constraints manipulation to destabilise current attractor states without using declarative instructions for body awareness	Developing dexterity or adaptiveness to constantly changing external information. Reducing the chances of choking under pressure through reinvestment of conscious control	(93)
*To ensure coupling to specifying (task-relevant) perceptual information*	Variability of practice	Using varied practice to ensure that information that is present in an environment but not reliable or specifying (such as distance for ball hitting), is not tightly attuned to, but instead through learning, more reliable information in the form of ‘time to contact’ is used instead	To support the attunement to specifying, rather than incidental, information during practice by ensuing that only specifying information is available in all practice environments	(94)
*Affording exploration of movements and perceptual landscapes*	Manipulating constraints to the task, environment, and organism	Using occlusion goggles to educate attention to more effective information sources such as more distal target related information such as the movement of other players. Encouraging a focus on outcome and finding multiple solutions	Constraints can be manipulated to change the available information sources—focusing education of attention to more specifying information. Setting up movement problems and asking learners to find a solution, then find a different one	(95, 96)
*To prevent injury caused by long-term technical errors within a movement*	Five-A model of technical refinement Using task constraints that highlight the movement form used	Using a process of analysis and bodily awareness to correct adverse elbow abduction in the weightlifting snatch movement Using a connection ball under the arm of baseball pitchers to highlight forearm flyout and give transitional feedback about changes to more effective movement solutions	Supports error detection and correction via a process of analysis, awareness, adjustment, (re)automation, and assurance Disrupts current movement solution and provides transitional feedback about the changes in movement solutions	(40–42, 97)
*When refining motor skills by altering biomechanics of movements that are already well established*	Facilitative somaesthetic awareness	Using internal focus verbal cues to “run with a flat foot” during gait retraining in running	Supports error correction and enables individuals to ‘relearn’ movements	(43, 46, 38)
*When developing broader psychological interventions to enhance self-efficacy or positive affect*	Using an external focus of attention combined with enhanced expectancies for success based on OPTIMAL theory of motor learning	Placing a cone to represent normative standing long jump data for individuals in the bottom 5th percentile in a standing long jump and directing individuals to try to jump as far past the cone as they can	Addresses the complex interaction between motivational and attentional factors that facilitate skill learning via goal-action coupling	(24, 25, 67)

Whilst these practice decisions may well be underpinned by competing theoretical approaches, an applied solution can still be found. For example, the benefits of a proximal attentional focus for novices can be underpinned by theoretical components of constrained action hypothesis (23) as well as Newell's (83) stages of skill acquisition (i.e., assembling a coordination pattern; gaining control and adaption of coordination; and skilled optimisation of coordination). In assembling coordination patterns, an individual is likely to need and use more proximal information. In the gaining control, during the stabilization phase, learning is focussed on attunement to specifying perceptual information, which can then be exploited in the skilled optimisation phase through effective calibration of action to the perceived information.

The principles of a constraints-led approach can be used to guide practice design that supports an education of focus of attention toward task-relevant information. These principles being: (a) goal orientated practice with clear session intentions; (b) manipulation of constraints to afford exploration of opportunities for action; (c) representative learning design that includes perceptual information that will be available in performance; and (d) repetition without repetition, encouraging the development of adaptable and effective movement solutions.

## Future research directions

Although the fundamental principle of adopting an internal or external focus of attention is simple, there remain ample avenues for future research. Above all else, the presently proposed ecological dynamics-based mechanisms for focus of attention effects are conjecture. However, so are information processing-based explanations until it is understood whether underlying neurophysiological mechanisms resemble information processing or ecological dynamics (98, 99). Out of the rather limited number of studies that have investigated the cortical processes underlying attentional foci, results suggest that internal foci of lesser task relevance may: (a) prevent visual inflow of environmental information to shield internal body-focused processing, via reductions in posterior alpha power (20, 100); (b) induce volitional control of attention to adjust behaviour responses to feedback via decreased frontal midline theta (101, 20); and (c) unbind muscles from a synergistic control strategy via reduced beta corticomuscular coherence between the contralateral motor cortex and effectors (20). Importantly, these neural mechanisms align with the proposals of ecological dynamics; the selective shielding/prioritisation of environmental vs. organismic constraints, cognition's supervision of attention to benefit perception for action, and binding/unbinding of synergistic control strategies, supporting the notion of an organism's self-organisation in response to its environment and task. Future research should continue to elucidate the neural mechanisms underlying both focus of attention and ecological dynamics, to inform theoretical understanding.

Another benefit of an ecological dynamics standpoint is its ability to account for results that are ill explained by common coding (57), constrained action hypothesis (23), or OPTIMAL theory (24, 25). Seminal literature's staunch advocacy of an external focus of attention (1) has resulted in comparatively little evaluation of instances where an external focus of attention may not be superior. However, noteworthy exceptions include appraisals of internal foci for somaesthetic awareness (38), a holistic focus of attention in instances without a clear external movement effect (28), and developmental benefits of adopting an internal focus of attention in proprioceptive sports [Olympic Weightlifting: (37)]. Overall, a body of literature is beginning to emerge which aligns with the concepts of ecological dynamics in suggesting that foci other than an external focus of attention can be facilitative. Future research should make concerted efforts to further understand applied and theoretical nuances within focus of attention.

With regards to applied nuances, the ecological dynamics-based framework has demonstrated itself popular within talent development research for its pertinent emphasis of multivariable effects [e.g., (102)]. It is proposed that no single independent factor can account for real-world differences in performance; instead, it is the combination of task (e.g., practice history), organism (e.g., anthropometrics and technical/tactical awareness), and environmental (e.g., relative age and sociocultural) constraints (73). Consequently, ecological dynamics offers a useful framework through which to investigate focus of attention effects observed in highly applied (i.e., ecologically valid) settings. For example, when comparing external vs. internal focus effects in a complex five-part gymnastics floor routine, assessed via the Federation Internationale de Gymnastique Code of Points, Lawrence et al. (26), observed no significant difference in performance based on attentional focus. In the absence of a more nuanced theoretical framework, null findings in the ecological study of Lawrence et al. were subsequently argued to be a product of methodological limitations (1, 27). However, it is possible that nuanced interactions between a multitude of variables meant that an internal focus of attention was able to yield unique benefits for participants. Going forward, ecological dynamics provides a promising framework for investigations in ecologically valid settings to avoid interpretational/publication bias.

Lastly, given doubts raised by recent research concerning an external focus’ ubiquitous superiority over an internal focus [e.g., (37, 38, 43)], it may be timely to re-evaluate what constitutes an optimal focus of attention, depending on skill and individual differences. To-date, investigations concerning possible foci of attention have been “top down” in their exploration of available foci; researchers traditionally identifying and prescribing the focus adopted by participants. Comparatively little research has attempted to utilise a “bottom up” approach [e.g., (36)], wherein optimal/preferred foci are identified by participants themselves. Such approaches may help identify further nuances to the focus of attention effect, in addition to distance (14), task relevance (19, 22), and breadth (50). Promising avenues to address this omission in current literature include think aloud protocols (103) and virtual reality (104). These methods respectively allow researchers to better assess and manipulate contextual information to ascertain novel nuances within the attentional foci adopted by participants.

## Conclusion

Literature surrounding focus of attention has come a long way since the original conception of internal and external labels by Wulf et al. (5). This initially binary choice has now expanded to encompass distance (14), relevance [e.g., (4)], salience [e.g., (26)], ecological validity [e.g., (37)], somaesthetic awareness [e.g., (38)], and wider psychological motivational factor considerations (24, 25). However, despite these advances in understanding attentional focus, theoretical explanations still rigidly advocate external foci [see (23, 24, 55)]. This is in stark contrast to the growing body of evidence demonstrating that external and internal foci of attention have distinct advantages depending on situational constraints. Accordingly, the presently proposed Ecological Dynamics Account of Attentional Focus is one of the first to provide a sufficiently flexible theoretical framework, which can explain instances of internal “and” external focus superiority. The implications of this are plentiful in facilitating more varied consideration of which focus may be optimal for a given scenario; research into both theoretical and applied aspects of the focus of attention phenomenon may just be getting started.
